# Recollections: Mainly of Artists and Writers

**Published:** 1985-07

**Authors:** Jack Davies

**Affiliations:** University Department of Pathology Bristol Royal Infirmary


**RECOLLECTIONS: MAINLY OF ARTISTS AND WRITERS**
**G. Grigson**
**Chatto & Windus: The Hogarth Press, London, 1984**
**pp. 195+ix, ISBN 0 7011 2791 0 Price £12.50**

Perhaps it is just as well that most medical practitioners are neither famous nor of any real interest to the general public. Professional writers, poor souls, are not spared the risks of fame. This book by Geoffrey Grigson spells out their problems. Fortunately none of us is likely to be the target (or subject) of such public critical appraisal. A tactfully phrased obituary notice in the medical journals is a more predictable fate. Have sympathy then, and yet enjoy the mixture of venom, understanding and the rhetoric.

Geoffrey Grigson is now eighty years old. His poems as a young man attracted much attention at the time. In later years his prose contributions have achieved the distinction of becoming collectors' items. Many of his occasional pieces have been gathered up in a variety of books, and are eagerly sought on booksellers' shelves.

This one is rather different. It is a compendium of reminiscences, making a personal Who-Was-Who in the literary 1930's. Lots of now respectable figures are there: T. S. Eliot, Sir Herbert Read, Henry Moore, Sir John Betjeman, and Sir Nikolaus Pevsner. Imagine the game. Suppose your own student contemporaries became not just respectable, but also transformed themselves into household names. How about tales of their early and less reputable days? This book carries much of that flavour.

That sort of thing and well written. Not necessarily nice, but great English. Try these thumb-nail assessments, unfair though they may be out of context:

Roy Campbell:

*Roy Campbell was—no Roy Campbell became, in middle age—an oaf.*

T. S. Eliot:

*I never heard T. S. Eliot laugh.*

Cyril Connolly:

*It does not do to name your animals after your friends . . . we owned a war-time pig, fattening slowly at the back of the garage . . . so we called him Cyril.*

Do not be put off. The reading is not just scurrilous. There are even some writers, notably Clere Parsons and Louis MacNeice, who escape with praise and are relatively unscathed. Other clearly have their oddities exposed in the reminiscences, often in snap-shot manner.

The prose of Grigson does however possess a curious character. Many times it seems intent on bursting into poetry, after breathlessly hurdling over a run of commas. This current collection is not the only one of Grigson's to display this leaping ability; he was as good at the trick in writing about Cornwall.[1]

The book is gripping; try it and see. I think we are much safer in medicine by not being of any interest to the general public. Why not stay in the auditorium; it is much safer and has its own entertainments.

Jack Davies
University Department of Pathology
Bristol Royal Infirmary

**REFERENCES**

1. GRIGSON, G. (1982) 'Freedom of the Parish'. *Anthony Mott, London,* No. 3 in the Cornish Library. (Reprint of 1954 publication by *Phoenix House Ltd.*). IBSN 0 907746 02 0.

